# Looking Through a Glass, Darkly: Eliminating Health Disparities

**Published:** 2006-06-15

**Authors:** Leandris C Liburd, H. Wayne Giles, George A. Mensah

**Affiliations:** Division of Adult and Community Health, Centers for Disease Control and Prevention; Division of Adult and Community Health, National Center for Chronic Disease Prevention and Health Promotion, Coordinating Center for Health Promotion, Centers for Disease Control and Prevention, Atlanta, Ga; National Center for Chronic Disease Prevention and Health Promotion, Coordinating Center for Health Promotion, Centers for Disease Control and Prevention, Atlanta, Ga

*It is critically important for policymakers to define the problem correctly so that our solutions address their intended goal — health security for all regardless of socioeconomic characteristics*.

William H. Frist (1)

*Although the determinants of health disparities are complex and varied, we do not need to unravel every last piece of this puzzle to begin to take action*.

Neil Calman (2)

Eliminating disparities in health status and access to health services for all U.S. populations is one of the two overarching goals of the *Healthy People 2010* national public health agenda, making it a top priority for the Centers for Disease Control and Prevention (CDC) and the public health community (3). Although health disparities have persisted in the United States for well over 100 years, we find ourselves in a historical moment when our nation can no longer tolerate this growing public health problem. The well-being of the entire nation is at stake. CDC's Racial and Ethnic Approaches to Community Health (REACH) 2010 is a cutting-edge, community-based public health program that is effectively eliminating health disparities in 40 communities of color across the nation. This issue of *Preventing Chronic Disease,* which features culturally appropriate, evidence-based strategies from REACH 2010 community projects, provides the opportunity for us to carry forward the research-to-practice wisdom shared during the 19th National Conference on Chronic Disease Prevention and Control, *Health Disparities: Progress, Challenges, and Opportunities*, held in March 2005.

Attracting more than 1600 public health workers, clinicians, and researchers to Atlanta, Ga, to critically examine our progress, challenges, and new opportunities to eliminate health disparities, the conference marked an important turning point in our longstanding commitment to eliminate health disparities. The opening performance by Sarah Jones —playwright, actress, and poet — brought the faces of health disparities into the conference and reminded all attendees that our deliberations were about real people confronting real challenges that affect their well-being and productivity every day. Throughout the conference, the energy and enthusiasm of the participants was high, the discourse was bold and forthright, and our commitment to achieving a nation without health disparities was sealed. The record attendance of lay community members, state and local public health leaders, academics, and representatives from private foundations and community-based institutions also signaled a national call to action to reverse the growing trend of health disparities. It is impossible to fully or fairly synthesize the information shared over the 3 days and more than 100 sessions of the conference, so we highlight here some of the major themes that emerged.

The facts about minority and other health disparities are pervasive and well documented. African Americans, Alaska Natives, American Indians, Asian Americans, Hispanic Americans, and Pacific Islanders are disproportionately affected by the leading causes of death and disability (4). We know that rates of immunization are lower among minorities (excluding Asian Americans, whose immunization rates are comparable to or better than whites) and that African American, American Indian, and Puerto Rican infants have higher death rates than white infants (4). Health care disparities among people living in Appalachia and other rural populations are linked to educational attainment, poverty level, and geography. For example, people living in rural Appalachia have the highest death rate from lung cancer and significantly higher death rates from cervical cancer than the overall U.S. population (5). Cardiovascular death rates for the rural Appalachian population are 19% to 21% higher than rates for the entire U.S. population (6). Without a coordinated, focused public health response, disparities in health will likely only worsen. Where to from here?

Even though the battle is far from being won, impressive improvements have been achieved in some key chronic disease indicators. In Alabama, for example, the REACH 2010 program, under the auspices of the University of Alabama at Birmingham, is making strides in eliminating disparities in breast cancer screening between African American and white women living in rural communities along the Black Belt. Unpublished data for Macon County, Alabama, indicate that the disparity in the use of mammography screening was reduced from 15% in 1998 to 8% in 2003 (M. N. Fouad, MD, MPH, 2006). According to the principal investigator, Dr Mona Fouad, "If the problem is in the community, then the solutions are in the community." She and her colleagues have built a strong base of community participation and support and credit the success of the program to their collaboration with more than 300 community volunteers.

Overcoming health disparities also demands attention to the social determinants of health, according to several expert panelists. They presented the results of research that sought to "unpack" the root causes of health care disparities among communities of color, rural communities, and other vulnerable Americans. Race, we were reminded, is a socially constructed concept and codes how racial and ethnic groups are positioned in the larger social structure as well as how resources are appropriated. Socioeconomic status (SES), although not well defined or consistently measured, is still a strong predictor of variations in health status (7). Typically, people of low SES fare worse than others. The reasons are multifactorial and intergenerational. On the other hand, contrary to popular belief, there is little evidence that high SES and health insurance coverage guarantee high-quality or equal care for people of color. What rethinking, then, needs to occur to eliminate discrimination in health care?

Several strategies to eliminate disparities in health care were put forth. Presenters called for the dismantling of separate and unequal access to health services through the formulation and enforcement of health policies that do not reward the selective displacement of patients in the health care system. Clinical researchers were urged to be more deliberate in recruiting and retaining people of color in clinical trials. Similarly, the need persists for more physicians, public health professionals, and other health care workers of color. The literature suggests that racial concordance between provider and patient has a positive effect on both the clinical encounter and patient adherence. The recruitment of the next generation of health professionals from communities of color, however, must begin during elementary school so that these students can later successfully compete for admission to college and then to graduate training programs. Panelists also argued that health care providers and community-based researchers must be culturally competent. Although the term *cultural competence* was contested among the presenters, the goal of cultural competency, simply put, is to acknowledge, understand, respect, and accommodate differences between providers and consumers of health care. Diversity exists both within and across the broad categories of racial and ethnic groups. Additional research is needed to assist providers and public health workers in understanding this diversity and its implications for clinical practice and public health program planning and implementation (8).

The Bronx Health REACH project has developed a seven-point advocacy agenda intended to eliminate the root causes of health disparities in the southwest Bronx, and the agenda mirrors the recommendations discussed at the conference. Namely, their coalition of health care providers and community and faith-based organizations are working to achieve "universal health insurance, an end to segregation in health facilities based on insurance status, accountability for state uncompensated care funds, culturally competent care for all, greater health workforce diversity, an expansion of public health education, and environmental justice" (2). The combination of broad community involvement, compelling data, and strong project leadership are advancing the Bronx Health REACH project toward its goals.

In this issue of *Preventing Chronic Disease*, REACH grantees report results from three communities — African American, Hispanic, and American Indian — addressing cardiovascular disease, type 2 diabetes, and associated risk factors such as sedentary lifestyles and poor nutrition. Community-based participatory research, multilevel intervention strategies, multiple settings, and the engagement of diverse community partners, including faith-based institutions, characterize the key components of these programs. Together, these grantees are producing knowledge that will guide the future direction of health disparities research and community-based interventions that will help us achieve our national overarching goal of eliminating health disparities.
